# Charcot–Marie‐Tooth type 2A in vivo models: Current updates

**DOI:** 10.1111/jcmm.18293

**Published:** 2024-05-09

**Authors:** Elena Abati, Mafalda Rizzuti, Alessia Anastasia, Giacomo Pietro Comi, Stefania Corti, Federica Rizzo

**Affiliations:** ^1^ Neurology Unit, Foundation IRCCS Ca' Granda Ospedale Maggiore Policlinico Milan Italy; ^2^ Department of Pathophysiology and Transplantation, Dino Ferrari Center Università degli Studi di Milano Milan Italy; ^3^ Neuromuscular and Rare Diseases Unit, Department of Neuroscience Fondazione IRCCS Ca' Granda Ospedale Maggiore Policlinico Milan Italy

**Keywords:** animal model, Charcot–Marie‐Tooth type 2A, mitofusin 2, mouse models

## Abstract

Charcot–Marie‐Tooth type 2A (CMT2A) is an inherited sensorimotor neuropathy associated with mutations within the *Mitofusin 2* (*MFN2*) gene. These mutations impair normal mitochondrial functioning via different mechanisms, disturbing the equilibrium between mitochondrial fusion and fission, of mitophagy and mitochondrial axonal transport. Although CMT2A disease causes a significant disability, no resolutive treatment for CMT2A patients to date. In this context, reliable experimental models are essential to precisely dissect the molecular mechanisms of disease and to devise effective therapeutic strategies. The most commonly used models are either in vitro or in vivo, and among the latter murine models are by far the most versatile and popular. Here, we critically revised the most relevant literature focused on the experimental models, providing an update on the mammalian models of CMT2A developed to date. We highlighted the different phenotypic, histopathological and molecular characteristics, and their use in translational studies for bringing potential therapies from the bench to the bedside. In addition, we discussed limitations of these models and perspectives for future improvement.

## 1 INTRODUCTION

Charcot–Marie‐Tooth (CMT) type 2A (CMT2A; OMIM 609260) is an inherited sensorimotor axonal neuropathy and is the most frequent subtype of axonal CMT.^1^, ^2^, ^3^, ^4^, ^5^, ^6^, ^7^ The disease is associated with mutations in the r *Mitofusin 2* (*MFN2*) gene, which encodes for the protein Mitofusin 2 (MFN2).^1^ MFN2 is a GTPase that is anchored to the outer mitochondrial membrane which is involved in the maintenance of the equilibrium between mitochondrial fusion and fission, as well as other mitochondrial processes.^3^ CMT2A is clinically defined by marked neuropathy, primarily affecting motor function, and in certain instances, accompanied by substantial proprioception loss. This results in progressive muscle weakness and atrophy in the legs and arms, typically onset during childhood and leading to considerable disability.^6^, ^7^, ^8^, ^9^ More than 100 *MFN2* mutations have been described in affected subjects,^10^, ^11^, ^12^, ^13^ Nevertheless, although CMT2A is generally associated with autosomal dominant or de novo dominant inheritance, few recessive and semidominant forms have been reported.^1^, ^2^, ^3^, ^5^, ^6^, ^7^, ^10^, ^11^, ^13^ MFN2 mutations seem to induce the disease through a ‘dominant‐negative’ mechanism, where the expression of the wild‐type (WT) MFN2 allele is negatively controlled by the mutant protein.^6^, ^14^, ^15^, ^16^, ^17^ To date, no clinical phenotype has been associated with certainty to *MFN2* variants resulting in haploinsufficiency. The molecular processes underlying neuronal degeneration associated with MFN2 mutations are not entirely clear. Recent advancements have shed light on the various mechanisms underlying MFN2‐related axonal degeneration, which include disruptions in mitochondrial transport and positioning^8^, ^18^, ^19^ as well as impairment in the communication between mitochondria and the endoplasmic reticulum.^20^, ^21^ Despite the efforts of researchers so far, no Food and Drug Administration (FDA) or European Medical Agency (EMA)‐approved curative or symptomatic treatments are currently available, and patients' management rely only on supportive care. The reasons for this lack of treatments, despite a known genetic basis, lie in several factors: firstly, the rarity of the disease, which makes it less appealing to both investors and the research community. In the second place, the absence of a clear gain‐of‐function or haploinsufficiency makes envisioning an effective curative strategy more complex. To precisely dissect and unravel the pathogenesis of CMT2A and to devise effective therapeutic strategies, reliable experimental models are essential. Here, we provide an overview and an update of the in vivo disease models with a focus on mouse models developed to date,^22^ highlighting similarities and differences of their phenotypic and molecular characterization and discussing limitations and perspectives for future improvement (Table 1).

**TABLE 1:** Comparison between available CMT2A mouse models.

	HB9 Mfn2*T105M^33^		Eno MFN2*R94Q^41^		Nestin‐cre MFN2T105M^46^		Thy1.2 MFN2R94Q^51^	Mfn2*K357T^64^		ENU‐induced MFN2*L643P^66^
Genotype	+/+	+/−	+/+ MitoCharc2	+/− MitoCharc1	+/+	+/−	Y‐linked	+/+	+/−	+/+
Transgene	Promoter: Hb9 Mutation: T105M		Promoter: Eno Mutation: R94Q		+/+ Promoter: Rosa‐STOP/CAG‐CreERT2 Mutation: T105M	+/− Promoter: Nestin‐Cre Mutation: T105M	Promoter: Thy1.2 Mutation: R94Q	Promoter: N/A Mutation: K357T		Promoter: N/A Mutation: L643P
Phenotype onset	Severe congenital	Mild congenital	Mild late	Mild late	6 weeks post‐tamoxifen induction	Mild late	Early	Postnatally letal	Mild late	Early severe
Motor performance	Hindlimb muscles weakness	No alteration	Frequent fall off at Rotarod test	Frequent fall off at Rotarod test	N/A	No alteration	Frequent fall off at Rotarod and Grip tests	N/A	No alteration	Decreased latency to fall at Rotarod and Grip tests
Gait	Defect in dorsi‐flexion but no gaiting alteration	No alteration	Abnormal print length	No alteration	N/A	Abnormal print length	Progressive gaiting worsening	N/A	N/A	Decreased locomotor activity and rearing events
Axon	40% fewer axons in motor roots	No alteration	55% increase of <3,5 μm axons	40% increase of <3,5 μm axons; Aδ fibres altered in the sciatic nerves	N/A	No alteration	Degeneration in tibialis muscle	N/A	No alteration	Reduced myelinated axons in the motor branch of the femoral nerve
Muscle fibre	Smaller anterior hindlimb muscles	No alteration	No alteration	No alteration	N/A	Smaller tibialis and soleus muscles	N/A	N/A	N/A	No alteration
Mitochondria	Highly aggregation and clusters	Highly aggregation and clusters	28% increase in mitochondria in <3,5 μm axons	34% increase in mitochondria in <3,5 μm axons	Reduced number in tibialis axons	No alteration	Clusters and morphology abnormalities. No mitophagy alteration	N/A	Clusters and morphology abnormalities in sciatic and optic nerves	Reduction in the average mitochondrial diameter
Sensoryphenotype	No alteration	No alteration	N/A	No alteration	N/A	No alteration	Precocious onset of severe sensorimotor symptoms	N/A	N/A	Impaired sensory responses

## 2 EXPERIMENTAL MODELS OF CMT2A

Experimental models of CMT2A have played a key role in elucidating underlying disease mechanisms. In addition, reliable models are essential to screen novel potential therapeutic compounds and to assess their efficacy and safety. In vitro works were performed first in non‐human primary cell lines (e.g. mouse embryonic fibroblasts (MEFs) and mouse dorsal root ganglia (DRGs))^3^ or in neuronal cell lines in which MFN2 function was silenced through RNA interference or modulated by overexpression of mutated MFN2.^14^, ^16^, ^23^, ^24^ These knock‐down or mutant cells show significant impairment of mitochondrial axonal transport and heightened mitochondrial clustering. Subsequently, induced pluripotent stem cells (iPSCs) derived from CMT2A subjects have been used to generate motor or sensory neurons with the same genetic background as donor patients,^18^, ^19^, ^25^, ^26^ offering a more accurate representation of the disease compared to previous models based on non‐human cell types or less disease‐relevant cell types (like fibroblasts). Overall, these works highlighted the role of mutated MFN2 in abnormal mitochondrial shape, motility, clustering and transport along the axons.

Concerning in vivo models, transgenic models in Drosophila and Zebrafish have proven invaluable in elucidating the role of MFN2 in mitochondrial dynamics and its contribution to the gradual decline of motor function.^27^, ^28^, ^29^, ^30^ The Drosophila model described by El Fissi et al.^31^ involved the incorporation of four CMT2A‐associated mutations (R364W, R94Q, T105M and L76P) into the *marf* gene, the Drosophila counterpart of the human *MFN2* gene. While these mutated flies exhibited comparable phenotypic and pathological aspects s, a notable distinction emerged when examining mitochondrial morphology. Clusters of unfused mitochondria were observed in mutations located within the GTPase domain (R94Q and T105M). The diminished fusion rate likely resulted from the loss of GTPase activity, while mitochondrial tethering leading to aggregate formation might have been triggered by unique features acquired by MFN2 mutants. This suggests a loss of function due to a dominant negative effect rather than haploinsufficiency. This finding in vivo supported the dominant negative effect of MFN2 mutants previously observed in DRG cultures.^14^ In contrast, R364W and L76P mutations induced opposing effects: the increased fusion rate resulted in large, round‐shaped mitochondria, suggesting that specific CMT2A mutations might have a dominant positive impact on MFN2 activity. In summary, this data demonstrated that both MFN2 loss and gain of function lead to disease traits in Drosophila models.^31^

Two groups evaluated the effects of MFN2 dysfunction in Zebrafish models. Vettori and colleagues generated a loss‐of‐function model using a knock‐down approach with a morpholino oligonucleotides (mfn2‐MO) directed against the acceptor splice site of intron 2 of *mfn2*, which introduces a premature stop codon.^28^ A control morpholino (nc‐MO) with no complementary RNA target was also concomitantly used. Knock‐down of mfn2 reduced the rate of survival of embryos, since 90% of nc‐MO‐injected embryos survived 24 h post‐fertilization, in contrast with only 60% of mfn2‐MO injected embryos. Mfn2 depletion also caused phenotypic alterations starting from 48 h post‐fertilization, which could be mild (curved tail, irregular somites, facial prognathism, underdeveloped eyes and brain ventricles edema) or severe (shortened body axis, little head with encephalic necrosis, ventral expansion of the yolk and trunk oedema) (mfn2‐MO vs. nc‐MO, *p* < 0.001). Mfn2 deficient larvae also displayed a significantly impaired escape response to physical touch (mfn2‐MO vs. nc‐MO, *p* < 0.001), indicative of motility alterations (mfn2‐MO vs. nc‐MO, *p* < 0.001). Immunofluorescence performed 48 h post‐fertilization revealed a significant difference in motor neuron (MN) organization between mfn2‐MO and nc‐MO treated embryos (*p* < 0.0001), together with a reduction of neuromuscular junctions (NMJs) was observed in mfn2‐MO‐treated embryos compared to nc‐MO controls.

Some years later, Chapman and colleagues published a study detailing a Zebrafish model carrying the nonsense mfn2 L285X mutation.^27^ They observed an adult and slowly progressive phenotype, with mfn2^L285X/L258X^ mutants displaying a significant progressive increase in mortality starting from day 175 (*p* < 0.0001) and a reduction of weight loss (*p* < 0.0001) and nose to tail length (*p* < 0.001) starting from day 60. Mutants also developed alterations in muscular function, that were tested by measuring critical swimming velocity in a manner similar to Rotarod test, through a swim tunnel apparatus which determines the maximum flow rate which fish could swim against for a sustained period. The results demonstrated a reduction of critical swimming velocity at 100 (mfn2^WT/WT^ vs. mfn2^L285X/L258X^, 28 cm/s vs. 12.11 ± 7.55 cm/s (mean ± SD), *p* < 0.01) and 200 (28 cm/s vs. 4.55 ± 3.23 cm/s, *p* < 0.001) days of life. At the same days, assessment of NMJs revealed an alteration in homozygous mutants compared with controls. The authors also reported a selective reduction in mitochondrial retrograde transport along the axons in mfn2^L285X/L258X^ neurons (*p* < 0.001).

Overall, these models provided the first evidence for a role of MFN2 in MN function and in mitochondrial axonal transport. Nevertheless, the complexity of mammalian physiology makes it difficult for them to faithfully replicate human disease.

## 3 MOUSE MODELS OF CMT2A

Mammalian models may offer a more pertinent approach to understanding human disease, although they come with physiological, genetic and technical constraints when attempting to model genetic neuropathy.^22^ A meaningful disease model relies on factors such as the mouse strain and its genetic background, which should not interfere with the observed traits, as well as the choice of a proper promoter used for the transgene delivery. Additionally, the development and assessment of such models are time‐consuming, and the attainment of the anticipated phenotype is not guaranteed.

So far, different murine disease models have been generated for CMT2A. Since KO of *mfn1* or *mfn2* resulted in murine embryonal death, possibly due to a placental defect,^3^ transgenic or mutagenized mice currently represent the most faithful in vivo disease models. A summary of available CMT2A mouse models is provided in Table 1.

### 3.1 HB9 mfn2^T105M^ transgenic model

In 2008, Detmer and his research team pioneered the creation of the first transgenic mouse model for CMT2A. They engineered this model to express the mfn2^T105M^ mutation, located within the GTPase G1 motif of MFN2, using the MN‐specific promoter HB9 which selectively target *mfn2* expression in transgenic embryonic mice spinal cord.^32^ HB9 is an MN‐specific promoter with proven activity in the developing and postnatal spinal cord, which holds two regulatory sequences in its distal region with a size of 313 and 125 base pairs that are sufficient for targeting gene expression exclusively to the spinal cord in transgenic embryonic mice.^33^ MFN2^T105M^ had been previously linked to early‐onset lower limb muscle atrophy, scoliosis and ataxia in unrelated CMT2A patient groups.^1^, ^34^ Intriguingly, MFN2^T105M^ protein correctly localized to mitochondria but exhibited defects in mitochondrial fusion and prompted the aggregation of mitochondria when in excess.^5^, ^16^ Given these findings in cell cultures, it was reasonable to anticipate that introducing these mutated *mfn2* alleles into mice's MNs could yield a reliable animal model replicating clinical motor impairments observed in CMT2A.

Heterozygous and homozygous Mfn2^T105M^ transgenic mice displayed varying levels of transgene expression, with heterozygous animals manifesting a notably less severe phenotype. This suggested that the phenotype was primarily influenced by the extent of transgene expression. Concerning motor symptoms, this model effectively mirrored some of the features characteristics in CMT2A patients. Homozygous transgenic mice exhibited critically deformed and substantially shorter tails, along with evident gait abnormalities from birth. Most of these animals (86%, *n* = 85) could not perform ankle dorsiflexion and showed hindlimb dragging when walking, reflecting tibialis anterior weakness, a typical clinical sign of CMT2A subjects, resulting in gait problems related to foot drop.^35^ Additionally, homozygous animals often presented clenched hind paws, seemingly unable to spread their toes. Nevertheless, homozygous mice managed to walk with short strides of their hindlimbs and performed adequately in rotarod and beam walking assessments. Differently from CMT2A patients, these hindlimb issues remained constant despite ageing.

Given that CMT2A patients typically exhibit distal muscle wasting in both the anterior and posterior compartments of their lower limbs, Detmer's group assessed this aspect in their model. Homozygous mice displayed a significant reduction in the muscle mass of the legs, particularly in the distal anterior regions, compared to wt and heterozygous mice; the muscle mass in the posterior hindlimb was similar among all genotypes. Microscopic examination of L4 and L5 motor roots revealed that homozygous transgenic animals had 40% fewer motor axons compared to wt mice, with more pronounced loss in the smaller‐calibre axons. Notably, in this model, the forelimbs remained unaffected, differently from CMT2A patients who often experience upper limb involvement in 50% of cases.^36^ In terms of sensory symptoms, both homozygous and heterozygous Mfn2^T105M^ transgenic mice did not display any sensory issues, even after 1 year of age.

Previously, ultrastructural examinations of the peripheral nerves of affected patients had disclosed anomalous mitochondrial clustering in the distal axon segments,^4^, ^37^, ^38^ accompanied by disruptions in the mitochondrial network and organization associated with MFN2 mutations.^16^, ^39^ Clustered mitochondria and irregular distribution along the axons were also seen in homozygous animals. It is plausible that this improper mitochondrial recruitment could contribute to axonopathy.

In summary, these findings underscore the significance of this initial mammalian model for replicating several, though not all, clinical aspects of the disease. Nevertheless, certain limitations exist, for instance, the presence of a pathological phenotype only in a homozygous genotype (while human disease shows a dominant pattern of transmission) and the restricted expression of the mutated MFN2 exclusively in MNs whereas in affected subjects, it is expressed ubiquitously.

### 3.2 Eno MFN2 ^R94Q^ transgenic model

In 2010, Cartoni and his group introduced a novel CMT2A transgenic mouse model named MitoCharc1 (heterozygous) and MitoCharc2 (homozygous), which carried the R94Q mutation in the human *MFN2* gene.^40^ This genetic alteration was controlled by a neuron‐specific enolase (*Eno*) promoter, ensuring widespread expression throughout the central nervous system (CNS) and peripheral nervous system (PNS).^41^, ^42^ Neuron‐specific enolase is a dimeric isoform of the glycolytic enzyme enolase found mainly in neurons. The 1.8‐kb genomic region upstream of the *Eno* gene is widely used as a neuron‐specific promoter.

For comparative purposes, they also generated Eno‐*MFN2* ^*WT*^ mice expressing the wt MFN2 (*MFN2* ^*WT*^) under the same promoter. At 5 months of age, both mutant lines displayed statistically significant locomotor deficiencies and gait abnormalities compared to MFN2^WT^ mice, as shown by the Rotarod test data (MitoCharc1 vs. MFN2^WT^, *p <* 0.001; and MitoCharc2 vs. MFN2^WT^, *p =* 0.001)t. Notably, the motor phenotype in MitoCharc1 animals appeared more consistent than that in MitoCharc2, possibly due to incomplete penetrance. MitoCharc2 mice exhibited even more pronounced phenotypic characteristics, including a lowered body posture, paw eversion and tail dragging, mirroring the walking difficulties and tendency to fall observed in CMT2A patients.^4^ Lately, Bernard‐Marissal's group deepened the characterization of this mouse model, performing multiple behavioural tests in MitoCharc1 mice at early (6 months) and late (12 months) time points.^43^ In addition to the confirmation of locomotor dysfunction via the Rotarod test, they found more comprehensive walking difficulties, with symptoms showing slight progression between 6 and 12 months. Specifically, MitoCharc1 mice exhibited alterations in paw pressure, contact surface, gait, posture and coordination as assessed by the CatWalk test (MitoCharc1 vs. MFN2^WT^, *p <* 0.05). Despite the locomotor impairments, no changes in muscle strength were found at either the 6‐ or 12‐month mark according to the grid test (6 months, WT: 2308 ± 96.2 and Mitocharc1: 2328 ± 144.8; 12 months, WT: 1886 ± 107.7 and Mitocharc1: 1852 ± 155.2). However, the model did demonstrate the loss of NMJs at 12 months of age, in absence of MN death. In terms of sensory function, this model did not exhibit significant impairments.^43^

Regarding other notable human disease features, such as abnormalities in mitochondrial structure and distribution,^4^, ^44^ 5‐month‐old MitoCharc1 mice showed an increase in number of mitochondria (roughly 30%), together with reduced mitochondrial diameters, in the distal portion of small calibre axons of the sciatic nerve. Bernard‐Marissal and his team also observed mitochondrial alterations in live imaging studies,^43^ revealing a lower proportion of moving mitochondria in the sciatic nerve of 1‐month‐old MitoCharc1 mice compared to WT (32% retrograde and 68% anterograde moving mitochondria in WT, 55% retrograde and 44% anterograde moving mitochondria in Mitocharc1). These morphological and speed changes in mitochondria could be attributed to reduced contacts between mitochondria and the endoplasmic reticulum (ER), particularly at the mitochondria‐associated membranes (MAM), as also observed in MNs' soma in the lumbar spinal cord of 12‐month‐old MitoCharc1 mice.^43^

Nerve conduction studies performed in MitoCharc1 mice showed alterations in the Aδ fibres component in the sciatic nerves, specifically an increase in the area/amplitude ratio of the compound action potential. This finding confirmed the observed axonal functional defects, which appeared to match the higher proportion of small to medium‐calibre axons, as the compound action potential area is influenced by the number and diameters of activated fibres.

In summary, Cartoni's mouse model seemed a significant advancement from the HB9 *mfn2* ^T105M^ transgenic model, as it expands *MFN2* transgene expression to the entire nervous system and replicates several characteristic disease symptoms, including locomotor impairment and axonopathy associated with changes in mitochondrial content and transport. However, a notable limitation of this model is the mild‐to‐late onset of pathological phenotypes, in contrast to the severe‐early clinical presentation typically seen in CMT2A patients with the R94Q MFN2 mutation.

### 3.3 Nestin‐cre MFN2^T105M^ transgenic model

In 2016, Bannerman and his team introduced two additional mouse models for CMT2A, both hemizygous and carrying the human T105M missense mutation in the *MFN2* gene.^45^ In a departure from previous models, Bannerman employed a knock‐in approach (Rosa‐STOP‐MFN2^T105M^) to extend the expression of the mutated *MFN2* transgene to all cell types.^46^, ^47^ Homozygous mice exhibited a broader clinical spectrum characterized by multiorgan dysfunction, severe motor function loss and the accumulation of abnormal mitochondria in Schwann cells within the sciatic nerve, which compromised their survival.

To address this issue, Bannerman and colleagues planned to restrict the expression of the *MFN2* transgene to neural and certain non‐neural cells, such as myosatellite cells, using the nestin‐cre strategy.^48^, ^49^ Given that a hallmark clinical feature of CMT2A patients is length‐dependent motor neuropathy, assessing variations in the muscle‐nerve interactions induced by mutant MFN2 is of great interest for translational applications.

Tests of MN function revealed no long‐term differences between hemizygous nestin‐cre MFN2^T105M^ mutant mice and control nestin‐cre mice, as evidenced by the normal resistance, equilibrium and coordination observed at the Rotarod test. However, the Noldus Catwalk system pointed out gait abnormalities, disclosing a statistically significant reduction in the print length of MFN2 mutant mice (control = 0.71 ± 0.072 cm, mutant = 0.58 ± 0.038 cm; *p* < 0.0003). These findings may be associated with one of the key CMT2A clinical signs, namely pes cavus.

These mice displayed a lower number of mitochondria in peripheral nerves, but they did not form clusters nor aggregates within MN soma, differently from findings reported in other models.^14^, ^16^, ^18^, ^19^ Additionally, the expression of the *MFN2* transgene did not alter axon numbers, sizes or g‐ratios. Nevertheless, it did lead to significant changes in muscle fibre diameter, particularly in the soleus and tibialis anterior muscles, which are involved in plantar flexion and dorsiflexion actions, respectively. Reduced expression of actin in the sarcomere, augmented levels of satellite cells and anomalies of striatal mitochondrial organization were also observed.

In summary, the nestin‐cre MFN2^T105M^ mouse model extends the expression of mutant MFN2 to all cell types, and presents critical phenotypic features of CMT2A patients, such as gait disturbances and reduction in the number of mitochondria in the tibialis muscle. On the contrary, certain findings appear to conflict with results from previous research works, and two limitations prevent it from being a suitable platform for drug testing, namely the late onset and low severity of symptoms and the absence of peripheral sensory impairment.

### 3.4 Thy1.2 MFN2^R94Q^ transgenic model

In 2019, Zhou and collaborators developed a new model of CMT2A, (Tg(Thy1‐MFN2R94Q)44Balo/J), characterized by hemizygous expression of the *MFN2‐R94Q* transgene under the thymocyte differentiation antigen 1, allele 2 (Thy1.2) promoter integrated into the Y chromosome.^50^ The murine Thy1.2 expression cassette derives from the mouse endogenous *Thy1.2* gene. Thy1.2, together with Thy1.1, is one of the two alleles of the *Thy1 membrane glycoprotein* gene. The *mThy1.2* expression cassette was engineered by eliminating all its exons, along with the second and third introns (which include the thymus‐specific enhancer). However, the first intron, housing the neural enhancer element, and the noncoding exons (Ia, Ib, II and IV) were preserved. The c.1070A > C mutation was then inserted within the MFN2WT coding sequence in the mThy1.2‐flag‐MFN2WT plasmid, in order to create the mThy1.2‐flag‐MFN2R94Q plasmid. The animals show symptoms and neuropathological characteristics with earlier onset and greater severity, thus making it a much more reliable model than the MitoCharc1 model, especially for studies evaluating the effectiveness of a therapeutic strategy. In particular, the Thy1.2‐MFN2^R94Q^ transgenic model shows signs of muscle weakness from the second month of life which become progressively more pronounced in the subsequent period in association with a reduction in visual capacity. Affected mice are significantly smaller than healthy pups and mortality is observed around 150 days of life. Due to the severity of the phenotype, Thy1.2‐MFN2^R94Q^ mice are not commercially available, while C57BL/6J‐Tg(Prnp‐MFN1)1Balo Tg(Thy1‐MFN2*R94Q)44Balo/ mice, which express in hemizygosis in addition to the Tg‐Th1MFN2‐R94Q also the Tg(Prnp‐MFN1) transgene under the control of the prion (Prnp) promoter in the CNS, are available for purchase. The overexpression of MFN1 through the Tg(Prnp‐MFN1) transgene in the nervous system improves the growth curve (at 5 months, Thy1.2‐MFN2^R94Q^ vs. Thy1.2‐MFN2^R94Q^:MFN1, *p* < 0.0001; WT vs. Thy1.2‐MFN2^R94Q^:MFN1, *ns*) and increases survival (Thy1.2‐MFN2^R94Q^ vs. Thy1.2‐MFN2^R94Q^:MFN1, *p* < 0.01; WT vs. Thy1.2‐MFN2^R94Q^:MFN1, *ns*) compared to mice hemizygous for the Thy1‐MFN2*R94Q transgene alone.

In the Cartoni mouse model, the Eno promoter ensures gene expression primarily in mature neurons, leading to the late appearance of neuromotor symptoms (after 5 months).^40^ Conversely, the transgene under the Thy1.2 promoter is expressed very early, from the first days after birth,^51^, ^52^, ^53^ and restricted to the CNS, targeting projection neurons, which are primarily affected in CMT2A patients.^54^ This promoter has been previously utilized in studies related to sensory nerve function and synaptic plasticity in both the CNS and PNS.^55^, ^56^, ^57^

Thy1.2‐MFN2^R94Q^ transgenic mice exhibited a significant reduction in body weight starting from the second month of life and reduced survival compared to nontransgenic and Thy1.2‐MFN2^WT^ mice. Behavioural assessments showed an impairment of neuromotor functions at the Rotarod test (at 6 months, Thy1.2‐MFN2^R94Q^ vs. WT, *p* < 0.0001) and grip strength test compared to WT or nontransgenic mice (at 6 months, Thy1.2‐MFN2^R94Q^ vs. WT, *p* < 0.0001; Thy1.2‐MFN2^R94Q^ vs. nTg, *p* < 0.0001 for both tests). In addition, sensory functions were also assessed. Visual acuity was measured using the optokinetic response test and optic nerve analysis, which revealed severe vision loss associated with significant reduction of neurofilaments and axonal spheroids.

Since some CMT2A patients demonstrate spinal cord and brain white matter changes,^9^, ^58^, ^59^ neuronal and axonal alterations in both the PNS and CNS were investigated in Thy1.2‐MFN2^R94Q^ mice, revealing axonal damage and loss in absence of neuronal death in the pyramidal tracts at the age of 5 months.^2^, ^60^, ^61^

On neuropathological analysis of nerve biopsies, aggregates of damaged mitochondria could be observed in the cytoplasm of neurons and along the axons of Thy1.2‐MFN2^R94Q^ mice. Similar observations have been reported in CMT2A patient nerve biopsies^4^, ^44^ and iPSC‐ and primary cell based in vitro models^5^, ^19^, ^24^ but have not been previously demonstrated in vivo.

Overall, this model accurately replicated crucial steps of CMT2A pathogenesis, including the precocious onset of severe sensorimotor symptoms, visual disturbances, diffuse axonal degenerations and mitochondrial abnormalities. It's worth noting that the disease phenotype was only limited to male mice due to the MFN2 transgene insertion into the Y chromosome, although no sex differences in phenotypes have been highlighted in clinical data from CMT2A patients thus far.

### 3.5 Mfn2^K357T^ transgenic model

In 2021, a new model carrying the previously undescribed p.K357T mutation found in a 4‐year‐old CMT boy was developed.^62^ The knock‐in mouse model was generated using the CRISPR/Cas9 genome editing and single‐stranded oligodeoxynucleotide (ssODN)‐mediated repair approach. Mfn2^K357T/K357T^ mouse pups did not survive after birth, while Mfn2^WT/K357T^ heterozygous mice showed significantly improved survival up to 10 months (Mfn2^K357T/K357T^ vs. Mfn2^WT/K357T^, *p* < 0.0001; Mfn2^K357T/K357T^ vs. Mfn2^WT/WT^, *p* < 0.0001), showing no symptoms and exhibiting normal histopathological features in their sciatic nerves. Voltage‐dependent anion channel 1 (VDAC1) is a protein located in the outer mitochondrial membrane. Immunofluorescence revealed VDAC1+ clusters in the spinal cords of Mfn2^K357T/K357T^ mouse pups, and, to a lesser degree, of Mfn2^WT/K357T^, suggesting aberrant mitochondrial clustering (Mfn2^K357T/K357T^ vs. Mfn2^WT/K357T^, *p* < 0.01; Mfn2^K357T/K357T^ vs. Mfn2^WT/WT^, *p* < 0.01; Mfn2^WT/K357T^ vs. Mfn2^WT/WT^, *ns*). Given the reduced survival of homozygous transgenic mice, subsequent analyses were performed only on hemizygous mice. VDAC+ clusters were also detected in the lumbar spinal cord white matter of 10‐month‐old Mfn2^WT/K357T^ mice (Mfn2^WT/K357T^ vs. Mfn2^WT/WT^, *P* < 0.05). Although histological and morphometric analyses revealed no differences between Mfn2^WT/K357T^ and Mfn2^WT/WT^ mice, ultrastructural analysis of sciatic and optic nerve axons of 8‐month‐old Mfn2^WT/K357T^ mice showed an altered distribution of axonal mitochondria with aberrant clustering and abnormal morphology, with disorganized or absent cristae, detachment of mitochondrial membranes and increased mitochondrial diameter.

Given the known anti‐inflammatory properties of MFN2,^63^ 6‐month‐old mice were exposed to lipopolysaccharide (LPS) in an attempt to uncover MFN2‐related changes following a systemic inflammatory stimulus. Four hours after LPS injection, Mfn2^WT/K357T^ mice exhibited a significant decline in the Rotarod test (Mfn2^WT/K357T^ *p < 0.05, **p < 0.01), and in the hindlimb grip strength test, while 48 and 96 h later, performances in the two tests did not differ significantly between the two groups. In addition, the levels of interleukin‐6 (IL‐6), a pro‐inflammatory cytokine, increased significantly in the serum of Mfn2^WT/K357T^ mice 4 h after LPS exposure (Mfn2^WT/K357T^ vs. Mfn2^WT/WT^, *p* < 0.0001), decreasing in the subsequent hours. The researchers also assessed CNS microgliosis 96 h after LPS exposure, showing that, in LPS‐treated Mfn2^WT/K357T^ mice, IBA1 intensity and area were significantly higher compared both to untreated hemizygous mice (Mfn2^WT/K357T^ vs. Mfn2^WT/WT^, *p* < 0.0001), and to Mfn2+/+ LPS mice at the level of the optic nerves (IBA1 fluorescence intensity: LPS Mfn2^WT/K357T^ vs. Mfn2^WT/K357TT^, *p* < 0.0001; LPS Mfn2^WT/K357T^ vs. LPS Mfn2^WT/WT^, *p* < 0.01; IBA1 area: LPS Mfn2^WT/K357T^ vs. Mfn2^WT/K357TT^, *p* < 0.01; LPS Mfn2^WT/K357T^ vs. LPS Mfn2^WT/WT^, *p* < 0.05) and lumbar spinal cord (IBA1 fluorescence intensity: LPS Mfn2^WT/K357T^ vs. Mfn2^WT/K357TT^, *p* < 0.0001; LPS Mfn2^WT/K357T^ vs. LPS Mfn2^WT/WT^, *p* < 0.01; IBA1 area: LPS Mfn2^WT/K357T^ vs. Mfn2^WT/K357TT^, *p* < 0.0001; LPS Mfn2^WT/K357T^ vs. LPS Mfn2^WT/WT^, *p* < 0.001). Analysis of cell infiltrates within the spinal cord revealed a significantly higher number of CD45+ (a general marker of overall cell infiltration) and CD68+ (macrophages) cells in the spinal cord of LPS‐treated Mfn2^WT/K357T^ mice compared to untreated hemizygous mice (cell number in whole lumbar spinal cord area, LPS Mfn2^WT/K357T^ vs. Mfn2^WT/K357TT^, CD45+ *p* < 0.0001, CD68+ *p* < 0.01) and to LPS‐treated WT mice (cell number in whole lumbar spinal cord area, LPS Mfn2^WT/K357T^ vs. LPS Mfn2^WT/WT^, CD45+ *p* < 0.001, CD68+ *p* < 0.05).

Overall, this model showed a late‐onset, subclinical phenotype with no overt disease signs. However, pathological analysis revealed aberrant mitochondrial clustering in their sciatic and optic nerves, altered mitochondrial morphology and localization and microglial activation in the CNS. Induction of stress with injection of LPS triggered a more severe peripheral inflammatory response and a significantly decreased motor performance 4 h after injection, as well as increased CNS infiltration and microglial activation after 96 h compared to treated WT mice.

### 3.6 ENU‐induced MFN2^L643P^ transgenic model

In 2023, Hines and colleagues published their study related to a novel *N*‐Ethyl‐*N*‐nitrosourea (ENU)‐induced mutant mouse model displaying a severe neuromuscular phenotype.^64^ ENU is a mutagen commonly used to generate mouse models of human diseases.^63^ Analysing the genomes of these mice can unveil the genetic mechanisms behind the observed disease features. This approach, termed forward genetic screening, is valuable for discovering novel disease‐related genes and understanding the biological pathways involved. Researchers at the Genomics Institute of the Novartis Research Foundation (GNF) utilized ENU‐induced mutagenesis, employing high‐throughput phenotyping methods to assess behavioural, metabolic and immunological characteristics. This effort led to the generation and identification of a mouse line carrying a homozygous mutation in exon 18 of *MFN2* (c.T1928C; p.L643P), within the highly conserved transmembrane domain. This mutation has not been described in patients so far, even though another missense mutation in the same transmembrane domain (p.R632W) was previously identified as potentially pathogenic.^65^ Behavioural evaluations on MFN2^L643P/L643P^ mice demonstrated progressive weight loss starting from 10 weeks of age and peaking at 16 weeks of age (MFN2^L643P/L643P^ vs. MFN2^WT/WT^, *p* < 0.0001 both females and males). They also showed a fast neuromuscular decline starting at 8 weeks of age, with decreased performance at the rotarod test (MFN2^L643P/L643P^ vs. MFN2^WT/WT^, *p* < 0.0001) and open field test (MFN2^L643P/L643P^ vs. MFN2^WT/WT^, distance travelled *p* < 0.0001, number of rearing events *p* < 0.01). Electrophysiological testing revealed no differences in motor and sensory NCV and CMAP amplitude in the sciatic nerve between affected and WT mice, but a significant increase to react to a wire filament poke or an elevated temperature stimulus in the Von Frey test and hot plate assay for mutant mice (Von Frey test: MFN2^L643P/L643P^ vs. MFN2^WT/WT^, *p* < 0.0001; hot plate assay: MFN2^L643P/L643P^ vs. MFN2^WT/WT^, *p* < 0.001), suggesting decreased sensitivity to mechanical stimulus and heat. Histopathological analysis of whole brains and lumbar spinal cords of mutant and WT mice at 12 weeks of age showed no gross abnormalities in cortical, midbrain or brainstem structures and no differences in the number of motor neurons in the lumbar spinal cord. Histopathological analysis of motor and sensory branches of the femoral nerve revealed a reduction in the number of myelinated axons in the motor branch (MFN2^L643P/L643P^ vs. MFN2^WT/WT^, *p* < 0.05). Histopathological analysis of gastrocnemius proved a slight, but nonsignificant increase in the number of partially innervated neuromuscular junctions and no signs of denervation, but ultrastructural assessment highlighted a significant reduction in the average mitochondrial diameter in mutant mice compared to WT (MFN2^L643P/L643P^ vs. MFN2^WT/WT^, *p* < 0.0001). Since reduced neuromuscular loading was shown to impair bone thickness and density, the authors analysed the tibiae and femurs of mutant and WT mice with micro computed tomography (CT), finding thinner bone cortex (MFN2^L643P/L643P^ vs. MFN2^WT/WT^, *p* < 0.05) and a reduced bone area fraction (MFN2^L643P/L643P^ vs. MFN2^WT/WT^, *p* < 0.05) in affected mice. These findings suggest potential defects in bone remodelling, in line with recent studies that demonstrated a role of Mfn2 in bone development.^66^, ^67^

Overall, the ENU‐induced MFN2^L643P^ transgenic model presented a recessive mild neuromuscular phenotype in mice, coupled with mild skeletal and mitochondrial abnormalities.

## 4 LIMITATIONS OF MURINE MODELS

The primary technical limitations of existing mouse models revolve around the level and pattern of expression of the *MFN2* gene, the causative factor in the disease. These models are transgenic, often involving the overexpression of mutant MFN2, typically through a human transgene. Some models restrict this expression to the CNS using promoters, while others have ubiquitous transgene expression. The critical question is whether a physiological level of expression of the mutant MFN2 is needed in order to accurately represent the pathogenic mechanism, or overexpression might be able to faithfully replicate it. This complex interplay between genetic expression, regulatory mechanisms, and the effect of a given mutation necessitates a nuanced approach when interpreting the results from animal models, highlighting the need for more refined and sophisticated methodologies to truly grasp the underlying mechanisms of neurological diseases.

In addition, it is worth pointing out that, among models reproducing autosomal dominant CMT2A, motor deficits were only observed in homozygous mice. Their heterozygous counterparts, with genetics similar to human patients, failed to develop a disease phenotype. This might be ascribed to differences between mice and humans in life span, disease duration and environmental exposure. It is also surprising that heterozygous mice displayed no disease progression or increasing severity during ageing. Such an observation raises several questions: is murine life expectancy too short to develop late‐onset neuropathy? Or is it possible that known pathogenic mutations act synergistically with other unknown genetic and epigenetic modifiers to accelerate or delay symptom onset? The genetic conundrum of CMT2A is exemplified by the wide phenotypic spectrum of human disease and by the impossibility to draw precise genotype–phenotype correlations. Indeed, the same mutations often result in different onset ages and disease severity. One open question is whether it is possible to develop an in vivo model that would collectively reflect all the features described in patients with various mutations. These findings notwithstanding, a unique mouse model overexpressing a single MFN2 mutation and with a severe, early‐onset phenotype might be enough to model the most severe hallmarks and as a platform for therapeutic assessment.

In the authors' experience, a more severe phenotype allows for easier testing of therapeutic compounds and stronger evidence of efficacy. In this perspective, the model Thy1.2‐MFN2^R94Q^ by Zhou and colleagues^50^ offers an early and severe sensorimotor phenotype and is commercially available, making it a potential choice for translational studies.

## 5 THERAPEUTIC IMPLICATIONS

No effective therapy has reached the clinic so far, although some strategies have obtained promising results in animal models.

Rocha and colleagues investigated mitofusin agonists, small molecules mimicking the MFN2 peptide–peptide interface, activating the protein allosterically and promoting mitochondrial fusion. Their study successfully reversed static mitochondrial clusters in cultured primary murine neurons carrying R94Q or T105M mutations and restored mitochondrial axonal transport in peripheral nerves of mice with the T105M mutation.^68^

Promising findings also emerged from studies involving histone deacetylase (HDAC) inhibitors, enzymes that deacetylate conserved serine residues in histones and nonhistone proteins. HDAC6, a cytoplasmic member, deacetylates alpha‐tubulin, a component of the cytoskeletal network. HDAC6 inhibitors demonstrated efficacy in HSPB1‐associated CMT2F transgenic mouse models, correcting axonal transport defects and reversing the CMT phenotype caused by HSPB1 mutations.^69^, ^70^, ^71^, ^72^ Following these results, HDAC6 inhibitors were tested in CMT2A models. A recent study explored the impact of pharmacological HDAC6 inhibition using the compound SW‐100 on MitoCharc1 mice. This treatment prevented α‐tubulin hypoacetylation and improved motor performance both at presymptomatic and postsymptomatic stages.^73^ The same research group also investigated new compounds derived from SW‐100, particularly SW‐101, that showed a significantly enhanced drug exposure in plasma and the brain in comparison with SW‐100. Administration of SW‐101 once daily to Mitocharc1 mice increased acetylated α‐tubulin levels in the distal sciatic nerve, counteracted progressive motor dysfunction, and ameliorated motor and sensory symptoms.^74^

Given that CMT2A primarily appears to be caused by a dominant negative effect rather than a toxic gain of function,^2^, ^6^, ^10^, ^13^ exploring the effectiveness of MFN2 overexpression alone is relevant because dominant‐negative activities can be overcome by the overexpression of wild‐type MFN2. Indeed, the overexpression of MFN2 also showed promising results in murine models. Increased expression of MFN2WT was indeed able to decrease the amount of mitochondrial aggregates induced by mutant MFN2 in a genetically modified primary neuronal cell line, and to rescue phenotypical and ultrastructural alterations in the Thy1.2‐MFN2R94Q mouse model.^50^

Recently, our group tested a new approach based on the combination of gene replacement and gene silencing via RNA interference (RNAi).^75^ In this study, both the mutant and wild‐type MFN2 mRNA are inhibited by a short hairpin RNA (shRNA), while the wild‐type protein is restored by overexpressing a cDNA encoding functional *MFN2* modified to be resistant to RNAi. We tested this approach in an in vitro IPSC‐derived motor neuronal CMT2A model^19^ and in the Mitocharc1 mouse model.^40^ In vitro, we observed a rescue of the cellular and molecular alterations in CMT2A MNs, more specifically we observed a reversal of altered mitochondrial distribution along the axons and a reduction of increased levels of mitophagy. We then delivered an AAV9 encoding the combination of shRNA/MFN2 cDNA (AAV9‐KD‐rMFN2) via intracerebroventricular injection in newborn Mitocharc1 mice, and observed a correct MFN2 molecular correction, with silencing of endogenous MFN2 and overexpression of exogenous MFN2 in their CNS. Further studies are needed to validate the therapeutic benefit of this strategy in animal models; however, this work provides a proof‐of‐principle of the feasibility of MFN2 silencing and replacement via AAV9 delivery in CMT2A mice.

On the contrary, also the overexpression of MFN1 would therapeutically benefit CMT2A models. Indeed, in vitro studies have demonstrated that MFN1 has the capacity to compensate for the loss of MFN2, safeguarding against mitochondrial fusion and transport defects induced by mutations in MFN2.^5^, ^76^ These data have been also confirmed in the Thy1.2‐MFN2R94Q transgenic mice expressing the point mutant R94Q on MFN2 specifically in neurons under the Thy1.2 promoter.^50^, ^77^ These mice recapitulate a range of neurological features observed in CMT2A. Notably, increased expression of MFN1 as a transgene (PrP‐MFN1) in the nervous system is well‐tolerated and results in nearly complete alleviation of phenotypic impairments in Thy1.2‐MFN2R94Q mice.^50^ Indeed, while mutant MFN2R94Q seems to initiate the tethering process with MFN1 or MFN2 on neighbouring mitochondria, it fails to execute full fusion, contributing to mitochondrial aggregation and to axonal degeneration. Furthermore, low levels of endogenous MFN1 in neurons promote a conformation of MFN2R94Q that allows MFN1‐MFN2 heterodimer tethering, enhancing the frequency of failed fusion events and mitochondrial clustering.

Overexpression of MFN1 in the Thy1.2‐MFN2^R94Q^ mouse model resulted in the normalization of the phenotype and of the transcriptomic signature and in the rescue of mitochondrial aggregation and axon degeneration. In a subsequent work using the same transgenic animals, Shahin and colleagues observed that increasing MFN1 levels successfully restored vision and retinal structure to normal levels by balancing the MFN1/MFN2 ratio and promoting PINK1‐dependent mitophagy.^78^ Thus, increasing MFN1 levels should restore fusion, improving the pathological phenotype.

Overall, these studies highlight the importance of animal models in the development of new therapeutic strategies.

## 6 CONCLUSIONS

**FIGURE 1:**
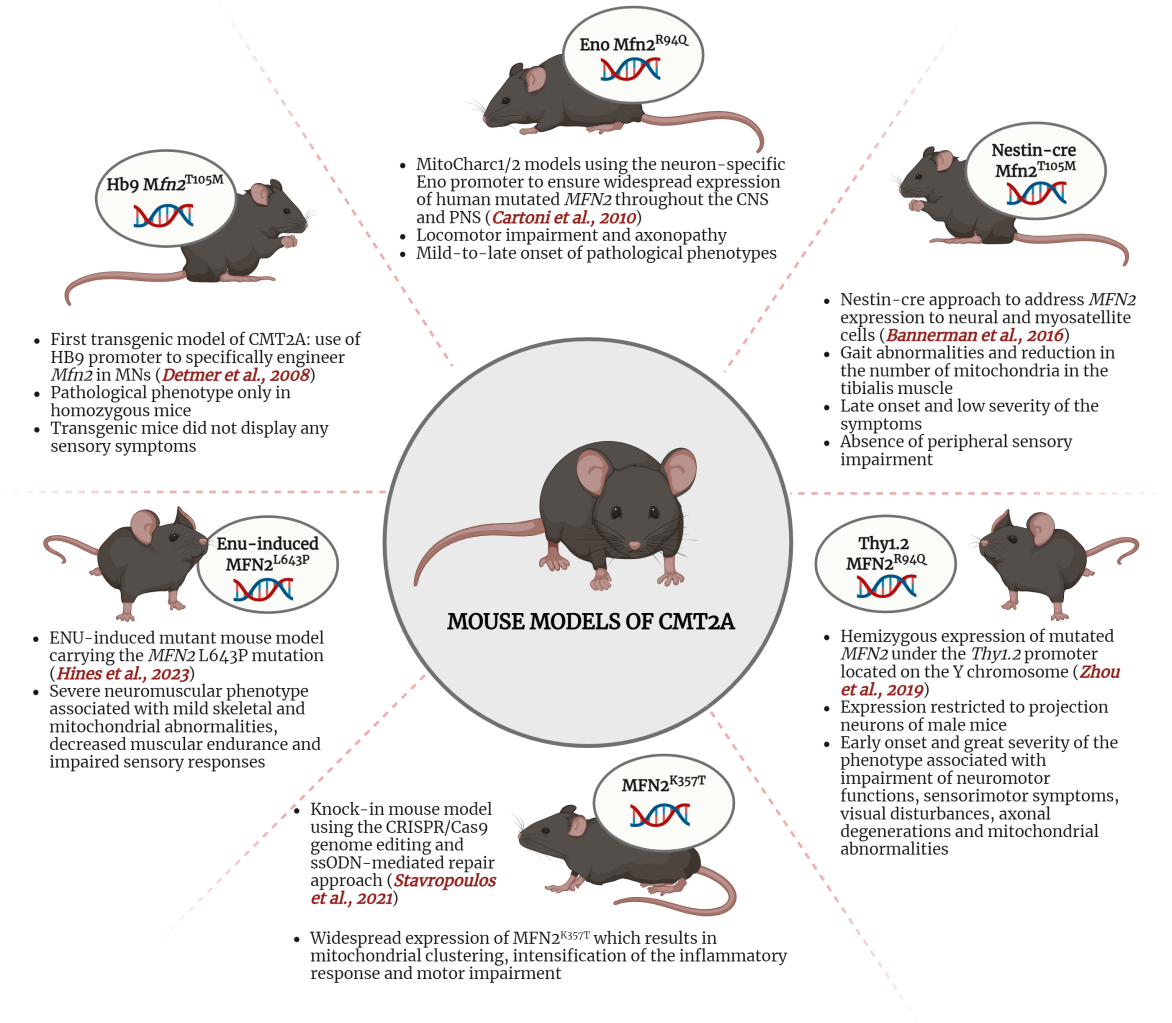
Schematic description of the characteristics of available CMT2A mouse models.

As mentioned above, CMT2A is a hereditary disease which causes a significant disability. Presently, there is no effective treatment which is able to revert or halt the progression of the underlying pathogenic mechanisms. Developing reliable animal models that accurately mimic genetic features and progress towards progressive, length‐dependent axonal neuropathy remains a significant challenge in understanding the disease and developing treatments. The existing mouse models mostly overexpress mutant MFN2 through a human transgene, some restricted to the nervous system via specific promoters while others exhibit ubiquitous expression (Figure 1). Questions persist about whether mimicking physiological mutant MFN2 levels is essential for understanding the disease mechanism and if humanized mice mirror patient regulatory mechanisms. The complexities of CMT2A, evidenced by diverse human clinical presentations and limited phenotype–genotype correlations, pose challenges in modelling mutations effectively. Integrating diverse model data—mice, humans, in vivo, in vitro—remains pivotal in constructing a reliable disease model. Despite the complexity, available models have provided vital insights into CMT2A, raising hope for the discovery and testing of potential therapeutic strategies.

## AUTHOR CONTRIBUTIONS

**Elena Abati:** Conceptualization (lead); investigation (lead); methodology (equal); project administration (equal); supervision (lead); writing – original draft (lead); writing – review and editing (equal). **Mafalda Rizzuti:** Conceptualization (equal); visualization (equal); writing – review and editing (equal). **Alessia Anastasia:** Data curation (equal); investigation (equal); writing – review and editing (equal). **Giacomo Pietro Comi:** Funding acquisition (equal); project administration (equal); resources (equal); supervision (equal); writing – review and editing (supporting). **Stefania Corti:** Funding acquisition (lead); investigation (equal); project administration (equal); resources (lead); supervision (equal); writing – review and editing (equal). **Federica Rizzo:** Conceptualization (equal); data curation (equal); formal analysis (equal); funding acquisition (lead); supervision (equal); writing – review and editing (equal).

## FUNDING INFORMATION

This study was funded by the Italian Ministry of Health grant GR‐2018‐12365358 to FR (2018–2021).

## CONFLICT OF INTEREST STATEMENT

The authors declare no conflict of interest.

## Data Availability

Data sharing is not applicable to this article as no new data were created or analysed in this study.
